# Characterization and Function Analysis of Soluble Dietary Fiber Obtained from Radish Pomace by Different Extraction Methods

**DOI:** 10.3390/molecules29020500

**Published:** 2024-01-19

**Authors:** Xiqian Tan, Xiaoxiao Cheng, Bingyu Ma, Fangchao Cui, Dangfeng Wang, Ronghu Shen, Xuepeng Li, Jianrong Li

**Affiliations:** 1 College of Food Science and Technology, Bohai University, Jinzhou 121013, China; tanxiqian@163.com (X.T.); chengxx0802@163.com (X.C.); mby9095@163.com (B.M.); cfc1031@163.com (F.C.); 15941611651@163.com (D.W.); 2 Hangzhou Xiaoshan Agriculture Development Co., Ltd., Xiaoshan, Hangzhou 311215, China

**Keywords:** soluble dietary fiber (SDF), fermentation, white radish, modification, extraction

## Abstract

Soluble dietary fiber (SDF) benefits human health, and different extraction methods might modify the structure and functions of the SDFs. Radish is rich in dietary fiber. To assess the impact of various extraction techniques on the properties and functions of radish SDF, the SDFs were obtained from white radish pomace using alkaline, ultrasonic-assisted, and fermentation-assisted extraction methods. Analysis was conducted on the structure, physicochemical characteristics, thermal properties, and functional attributes of the SDFs. The study revealed that various extraction techniques can impact the monosaccharides composition and functionality of the SDFs. Compared with the other two extraction methods, the surface structures of SDFs obtained by fermentation-assisted extraction were looser and more porous, and the SDF had better water solubility and water/oil holding capacity. The adsorption capacities of glucose and cholesterol of the SDFs obtained from fermentation-assisted extraction were also improved. *Wickerhamomyces anomalus* YFJ252 seems the most appropriate strain to ferment white radish pomace to acquire SDF; the water holding, oil holding, glucose absorption capacity, and cholesterol absorption capacity at pH 2 and pH 7 have a 3.06, 1.65, 3.19, 1.27, and 1.83 fold increase than the SDF extracted through alkaline extraction method.

## 1. Introduction

Dietary fiber (DF) is abundant in many fruits and vegetables. Based on their solubility, dietary fibers can be categorized into soluble dietary fiber (SDF) and insoluble dietary fiber (IDF) [1]. SDF contains soluble hemicellulose, gums, plant mucilage, and pectin, while IDF contains lignin, cellulose, chitosan, and intractable hemicellulose. Due to its prospective physicochemical characteristics and functional properties, SDF has received greater attention than IDF [2]. SDF could delay the progression of obesity by increasing fecal bulk, reducing transit time, helping lower fat accumulation, improving the serum lipid profile, and increasing basal metabolism [3]. SDF are rapidly fermented, while IDF are slowly or only partially fermented [4]. They were also regarded as a significant nutrient to the fiber-degrading bacteria in the gut [5] and could increase the diversity of the gut microbiota and the colonization of beneficial bacteria. Research proved that SDF could decrease the ratio of *Firmicutes*/*Bacteroidetes* and increase the relative abundance of the genera *Roseburia* [6]; it was further found certain SDF could enrich probiotic bacteria, such as *Akkermansia*, *Bifidobacterium*, *Faecalibacterium*, and *Prevotella* [7].

Radish (*Raphanus sativus* L.), a member of the Brassicaceae family, is an excellent source of nutrients, including flavonoids, vitamins, minerals, dietary fiber, amino acids, and glucosinolates [8], which has antioxidative, antibacterial, anti-inflammatory, lipid-lowering, kidney-protective, and anticancer properties [9]. The processing of radish might generate some by-products, such as pomace, which still contains a wealth of bioactive compounds and direct disposal of the radish by-products results in economic loss and pollution of the environment. The recovery and utilization of by-products from agricultural and industrial production is a trend for current research [10,11]; a series of studies have extracted SDF exhibited different functions from fruit or vegetable pomace such as grapefruit peel [12], Litchi pomace [13], tomato pomace [14], and potato pomace [15]. The physiochemical and biological functions of the SDF might vary among different food matrices. Research performed on SDF obtained from defatted rice bran through *Trichoderma viride* fermentation found that it exhibited higher water solubility (WS), water/oil-holding capacity (WHC/OHC), and cholesterol absorption capacity (CAC) [16]. However, the structures and functions of SDFs extracted from the radish pomace have not been thoroughly investigated.

The extraction methods of SDF include chemical (acid, alkali), physical, and biological methods. Different extraction methods are known to alter the structural and functional properties of the SDFs [17,18]. Given this, it is important to contemplate how to generate superior dietary fiber and enhance the physiological efficacy of dietary fiber derived from radish. Microbial fermentation is a secure, effective, and economical approach for producing and enhancing premium dietary fiber [19]. Fermentation enhances the SDF content in fermented food products by the impact of enzymes and the acidic environment on food matrices. Due to the increase in solubility and multiple surface-active groups after fermentation, the functions of the SDFs might be improved [20]. β-glucosidase (EC3.2.1.21) is part of the cellulose family and plays a role in the synthesis and hydrolysis of substances by carrying out glycosylation and deglycosylation reactions. These processes include breaking the bonds between non-carbohydrate and carbohydrate components of the substrates. β-glucosidase could be isolated from different microorganisms and exhibited sophisticated enzyme capacities [21]. Research has proved that fermentation through β-glucosidase producing lactic acid bacteria could increase the SDF content [22].

In our study, the SDF of radish pomace was extracted using alkali, ultrasound-assisted, and fermentation-assisted extraction methods. The effects of different extraction methods, especially the fermentation-assisted extraction method, on the monosaccharide compositions, structures, and functional properties of radish SDFs were determined. Three strains, *Lactobacillus plantarum* C11, *Bacillus amyloliquefaciens* HC2, *Wickerhamomyces anomalus* YFJ252, which were isolated from different sources by our laboratory and all have β-glucosidase producing abilities, were used in the fermentation of radish pomace to modify its SDF. This study could provide ideas and offer valuable insights into the utilization of SDF derived from radish pomace as a functional food ingredient.

## 2. Results and Discussion

### 2.1. Spectral Analysis of SDF

**Figure 1:**
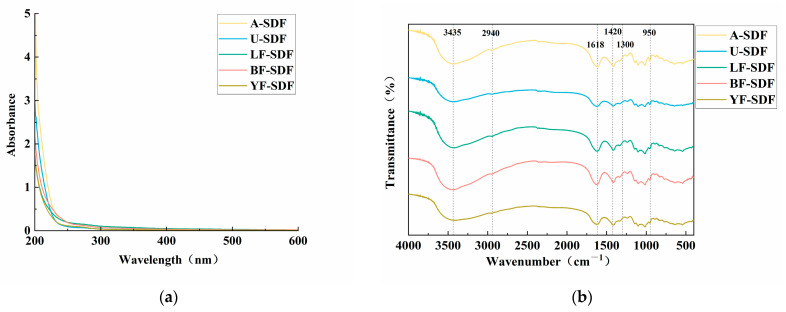
The spectral analysis of the SDFs. (**a**) UV spectra; (**b**) FT-IR spectra.

The SDFs extracted by different extraction methods were designated as A-SDF (alkali extraction), U-SDF (ultrasound-assisted extraction), LF-SDF (*L. plantarum* C11 fermentation-assisted extraction), BF-SDF (*B. amyloliquefaciens* HC2 fermentation-assisted extraction), and YF-SDF (*W. anomalus* YFJ252 fermentation-assisted extraction) (the detailed explanation of the extraction method could be found in Section 3.2). The UV spectra of SDFs extracted by different methods are shown in Figure 1a. There were no characteristic absorption peaks at 260 nm and 280 nm for all the SDFs extracted by different methods, indicating no nucleic acids and water-soluble proteins existed in the extracted SDFs.

The Fourier-transform infrared (FT-IR) results are displayed in Figure 1b. The prominent and wide peak observed in the 3750–3000 cm^−1^ range is ascribed to the stretching vibration of O-H bonds in cellulose and hemicellulose [23]. The peaks observed at 2943, 2939, and 2941 cm^−1^ are attributed to the stretching vibration of C-H groups. These peaks are commonly observed in polysaccharide-based polymers and serve as characteristic markers. The signal at 1600 cm^−1^ signifies the distinct absorption of the C=O link in uronic acids, suggesting a greater concentration of uronic acids in the monosaccharide components [24]. The 1403 cm^−1^ region exhibits the stretching vibration of -CH_3_ groups. The absorption peak at 1420 cm^−1^ is associated with the absorption of crystalline regions. The graph shows that this peak is considerably diminished in the U-SDF group, demonstrating a decrease in crystallinity caused by the ultra-sound treatment [25]. The spectral range from 1300 to 950 cm^−1^ is called the “fingerprint region” of polysaccharides. It corresponds to the stretching vibration of C-O-C and C-O-H bonds [26]. The findings revealed subtle variations in the composition of SDFs acquired using various extraction techniques.

### 2.2. Monosaccharide Composition of the SDF

The monosaccharide composition of the SDF is shown in Table 1 and Figure S1 in the Supplementary Materials. It was shown that A-SDF, U-SDF, LF-SDF, BF-SDF, and YF-SDF are mainly composed of glucose, mannose, rhamnose, galactose, xylose, arabinose, fucose, ribose, glucuronic acid and galacturonic acid, at different molar ratios. The presence of galacturonic acid is the main component for all the SDFs, U-SDF had the highest content of galacturonic acid (17.84%), followed by BF-SDF (12.99%), YF-SDF (10.30%), A-SDF (10.21%) and LF-SDF (8.37%). The results mean that all the SDFs were acidic polysaccharides, and the main ingredient of the SDF samples was pectin [27], which might be due to the hydrolysis of hemicellulose or cellulose. YF-SDF has the highest content of mannose and rhamnose, at 4.49% and 4.73%, respectively. It was confirmed that the existence of mannose and rhamnose could improve the antioxidant and hypoglycemic activity of the SDFs [28]. Arabinose plays the role of modifying the xylan main chain of the hemicellulose polysaccharides. It is proved that arabinose-rich polysaccharides exhibited superiority in immunostimulatory activity [29]. The existence of arabinose would be related to the function of radish SDFs. The results indicated the monosaccharide composition of radish SDFs was altered by different extraction methods and the fermentation of different strains, and the monosaccharide composition would respond to the function of the radish SDFs.

### 2.3. Particle Size and Surface Morphology

**Figure 2:**
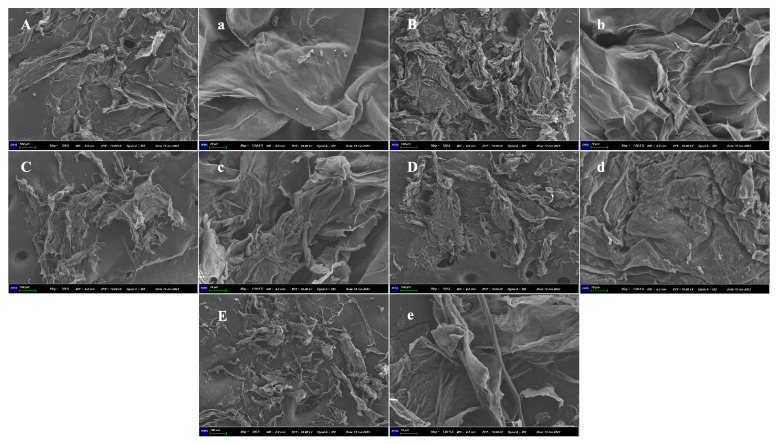
SEM micrographs of SDF. A-SDF (**A**): 100×, (**a**): 1000×; U-SDF (**B**): 100×, (**b**): 1000×; LF-SDF (**C**): 100×, (**c**): 1000×; BF-SDF (**D**): 100×, (**d**): 1000×; YF-SDF (**E**): 100×, (**e**): 1000×.

The particle sizes of the A-SDF, U-SDF, LF-SDF, BF-SDF, and YF-SDF were 954.07 ± 156.77, 461.51 ± 13.36, 697.32 ± 55.22, 134.84 ± 59.92, and 418.71 ± 113.33 nm, respectively. Compared with A-SDF, the particle size of U-SDF, BF-SDF, and YF-SDF decreased significantly (*p* < 0.05), which was due to the loose surface structure of SDF caused by ultrasonic treatment and fermentation treatment (Figure 2), thus reducing the average particle size of SDF.

Scanning electron microscopy (SEM) is used to explore the microstructure of SDFs. As shown in Figure 2, the surface of A-SDF and U-SDF exhibited more compacted structures with less pore, and loosening than LF-SDF, BF-SDF, and YF-SDF. The surface of A-SDF was smooth with no cavities, showing a dense, silk-like flat parcel. Fermentation increased the irregularity of SDF microstructures, leading to severe impairment and loosening surface morphology. The SDF extracted through the fermentation-assisted extraction appeared wrinkled, curly, and loose in appearance. Research proved that loose spatial structures exhibit increases in specific surface areas, which might influence the adsorption capacities of oil, water, and glucose [17]. The network or loose structure could expose more polar groups and binding sites of the SDFs, hence improving their functional value [30]. Therefore, based on SEM results, it could be inferred that the structures of the radish SDF undergo alteration depending on the extraction method.

### 2.4. X-ray Diffraction (XRD) Analysis

**Figure 3:**
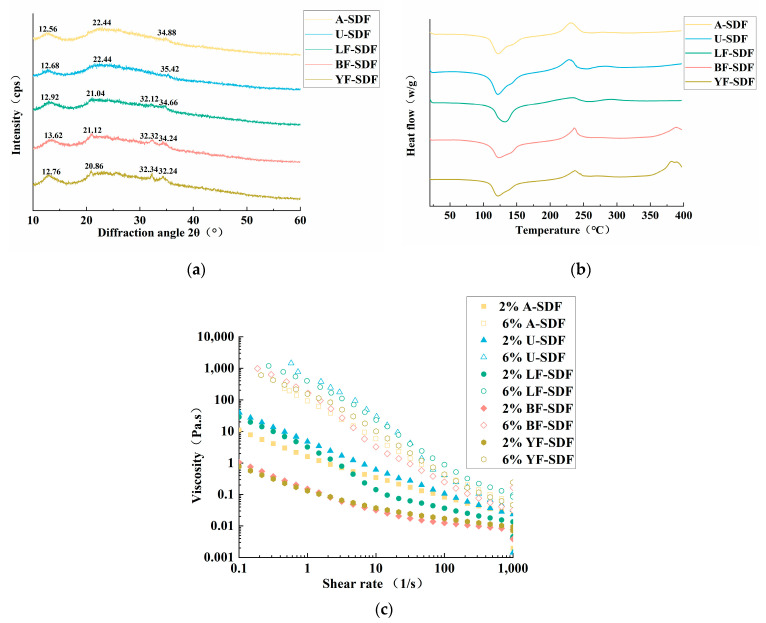
Physicochemical properties of SDF. (**a**) XRD analysis; (**b**) DSC analysis; (**c**) Rheological properties.

XRD is used to determine the crystalline properties of SDFs (Figure 3a). The XRD patterns of the SDFs were similar, with a characteristic gentle and broad diffraction peak at 20.82° 2θ indicating that they were the main substances of amorphous structure in the crystal region, which was similar to the crystal structure of type I cellulose [18].

### 2.5. Differential Scanning Calorimetry (DSC) Analysis

The thermal stability of SDFs was assessed by DSC. As shown in Figure 3b. The first significant heat absorption peak appeared at 100–150 °C, possibly due to the change of pectin from crystalline to amorphous structure and water evaporation. The second peak appears at about 230 °C, corresponding to the peak of pectin exothermic heat [31]. The obvious exothermic peaks between 225 °C and 250 °C are caused by the thermal decomposition and oxidative decomposition of the polymer [32]. BF-SDF and YF-SDF also showed endothermic peaks between 350 °C and 400 °C. It is because of the thermal decomposition of cellulose, hemicellulose, and lignin. In addition, the higher the thermal strength of the sample, the lower the thermal stability; hence, LF-SDF has higher thermal stability than the other SDF.

### 2.6. Rheological Properties

As shown in Figure 3c, the shear rate of the SDFs decreased gradually at room temperature, presenting a shear dilution phenomenon, which showed that SDFs belonged to pseudoplastic non-Newtonian fluids [33]. With the increase in concentration, the viscosities of SDFs increased accordingly. That might be because the interaction force among the SDF molecules was elevated at high concentrations, which increased the degree of cross-linking and polymerization of substances. Moreover, at the same concentration and shear rate, the highest viscosity of 2% concentration of the SDF was U-SDF, followed by LF-SDF, A-SDF, BF-SDF, and YF-SDF, the highest viscosity of 6% concentration of the SDF was U-SDF, followed by LF-SDF, YF-SDF, A-SDF, and BF-SDF. The high viscosity of U-SDF of different concentrations might be associated with compositions of pectin, which is coordinated with the results of monosaccharide compositions. Prior research has demonstrated that viscosity is a significant characteristic of SDF, and it is strongly linked to SDF’s capacity to slow down and diminish the absorption of meal intake in the digestive system, hence reducing the postprandial blood glucose response [13].

### 2.7. Functional Properties of SDF

#### 2.7.1. Water Solubility (WS), Water Holding Capacity (WHC), and Oil Holding Capacity (OHC)

**Figure 4:**
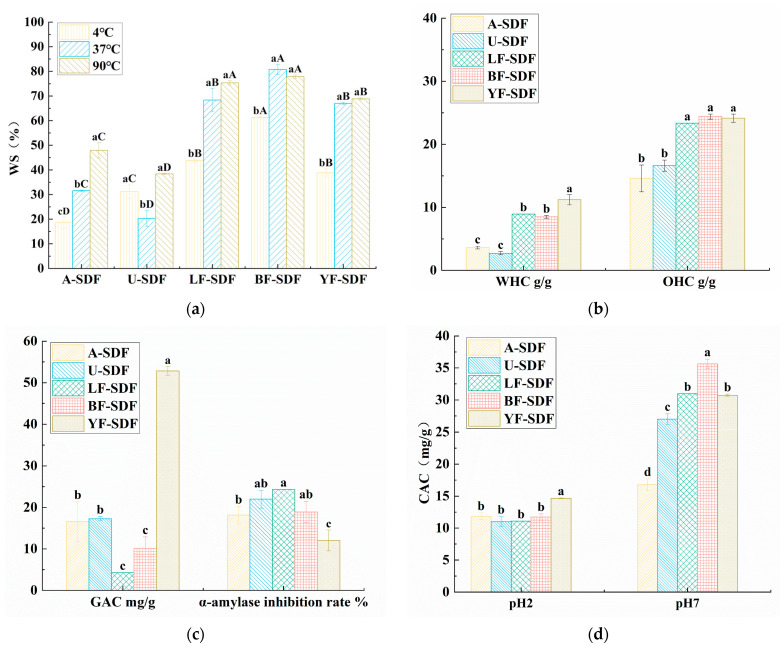
Functional properties of SDF. (**a**) WS capacity; (**b**) WHC and OHC capacity; (**c**) Glucose adsorption capacity and α-amylase inhibition ability; (**d**) Cholesterol adsorption capacity. Results were obtained from three experimental replicates (*n* = 3). Different uppercase letters (A–D) and lowercase letters (a–d) indicate statistically significant differences between and within SDF groups, respectively (*p <* 0.05). Statistical analysis was carried out using ANOVA analysis followed by Ducan’s test.

Figure 4a analyzed the WS of SDFs at 4, 37, and 90 °C. The WS of BF-SDF was higher than the WS of the other SDFs, LF-SDF and YF-SDF also have better WS than A-SDF and U-SDF. For A-SDF, its WS was significantly different (*p* < 0.05) at different temperatures, it had the highest WS at 90 °C (47.90 ± 3.10%), and the lowest WS at 4 °C (18.60 ± 2.40%), the unstable WS of A-SDF might restrict its use in the food industry. For LF-SDF, BF-SDF, and YF-SDF, their WS at 37 and 90 °C were significantly (*p* < 0.05) higher than that at 4 °C, and there were no significant differences for the WS of these SDFs at 37 and 90 °C. The WS ability of different SDFs might relate to the loose and porous structure, and perhaps more polar groups were exposed on the surface of SDF after fermentation [20], which is in line with the SEM results.

The water and oil holding capacities are presented in Figure 4b. WHC refers to the ability of wet materials to retain water under external force. The results show that YF-SDF has the highest WHC (11.2 ± 0.8 g/g), and there were no significant differences among LF-SDF, BF-SDF, A-SDF, and U-SDF, which means the *W. anomalus* YFJ252 fermentation-assisted extraction could achieve SDF with better WHC. The WHC and OHC properties of dietary fiber are closely related to its source, structural morphology, porosity, and extraction methods. Fermentation can break down large macromolecular compounds, such as insoluble dietary fiber (IDF), into smaller molecular components, such as the fermentation of citrus dregs by *Penicillium* sp. Cis16 improved the yield of SDF from 6.77% to 36.56% [34]. Additionally, it can enhance the specific surface area of SDF and generate porous and vacant structures, promoting the emergence of additional polar groups and facilitating the formation of more hydrogen bonds or dipoles with water. And our research proved that the effect of fermentation seems to be related to the strains.

The oil-binding property of dietary fiber is crucial in food applications, such as preventing fat loss during cooking. Additionally, it denotes how dietary fiber can physically bind and retain oil or fat, decreasing its absorption throughout food digestion. The OHC of SDF is contingent upon the specific characteristics of the fiber, including its type, qualities, and the procedures used for extraction. The OHC of YF-SDF, BF-SDF, and LF-SDF were 24.15 ± 0.65, 23.4 ± 0.40, 23.35 ± 0.25 g/g, respectively, there was no significant difference (*p* > 0.05) among the LF-SDF, BF-SDF, and YF-SDF, at the same time there was no significant difference (*p* > 0.05) between A-SDF (14.6 ± 2.13 g/g) and U-SDF (16.63 ± 0.88 g/g), however, the OHC of the SDFs extracted by fermentation-assisted extraction were significantly higher (*p* < 0.05) than those extracted by alkali and ultrasound-assisted extraction. The SDFs in the fermented group were more potent than the SDFs extracted by alkali and ultrasound. As the SEM findings indicated, this could be attributed to its permeable and unconsolidated composition. The elevated OHC of dietary fiber could potentially impact the assimilation of dietary lipids within the gastrointestinal tract, hence aiding in maintaining physical fitness and stabilizing blood lipid levels. Previous studies have demonstrated that a promising extraction method, ultrasound-assisted deep eutectic solvents method, could obtain DF from navel orange peel of (7.78 ± 0.29 g/g), which is significantly higher than the enzymatic extraction method [35], which proved that enzymatic extraction might not be suitable for the extraction of SDFs from plant residue, and the results of our study elucidated that fermentation might be superior to ultrasound-assisted methods. The SDFs with high viscosity, WHC and OHC could be used as additives in product formulation. For instance, it could replace the fat in hazelnut spread creams [36].

#### 2.7.2. Glucose Absorption Capacity (GAC) and the Inhibition Capacities of α-Amylase

The GAC of different SDFs extracted using different methods are listed in Figure 4c. YF-SDF has the highest GAC and was 52.84 ± 1.04 mg/g, which was significantly higher than the other SDFs (*p* < 0.05); the GAC of SDFs obtained by fermentation-assisted extraction are also greater than of A-SDF and U-SDF. Interestingly, although the GAC of YF-SDF was 3-fold greater than A-SDF and U-SDF, the GAC of LF-SDF and BF-LDF have no significant difference, as do the A-SDF and U-SDF. It is indicated that the GAC of the SDF obtained by fermentation-assisted extraction might be strain-specific, and might also be related to the food matrix. Some research has proved that fermentation could improve the GAC of the extracted SDF. It was proved that SDF extracted from the fermented *R. roxburghii* pomace fermented with *Bacillus natto* could also have a 1.4-fold improvement of GAC compared to the SDF extracted without fermentation [19]. Another research confirmed superfine grinding combined with *L.Papacasei* fermentation-modified Dabumiyou peel could significantly improve the GAC of the SDFs compared with the other grapefruit (Shatianyou, Majiayou, and Jinggangmiyou) [37]. Hence, the results of our study proved that *W.anomalus* YFJ252 assisted fermentation could be a better choice to extract SDFs from radishes with higher GAC.

The increases in blood glucose and glucose absorption after meals are related to starch digestion, and α-amylase is one of the key enzymes in starch decomposition and absorption [38]. SDF can reduce starch digestion by inhibiting α-amylase activity, thus reducing glucose release [39]. Therefore, inhibition of α-amylase activity effectively alleviates the rise of blood glucose caused by starch digestion after meals. As shown in Figure 4c, the inhibitory rates of α-amylase of LF-SDF, BF-SDF and U-SDF, which were 24.33 ± 2.66, 18.89 ± 2.6, and 21.96 ± 2.16%, respectively, were higher than those of A-SDF and YF-SDF, which were 18.12 ± 2.11 and 12.04 ± 2.52%, respectively. Research has shown that SDF can inhibit the activity of α-amylase by altering the structure of the enzyme and preventing the binding of substrates to the active site of the enzyme [40].

#### 2.7.3. Cholesterol Absorption Capacity (CAC)

Cholesterol is a crucial nutrient that is both a necessary component of the body’s structure and a building block for generating important compounds. Reports indicate that augmenting the intake of SDF (soluble dietary fiber) will effectively lower cholesterol levels [41]. Therefore, under the simulated gastric and intestinal conditions of pH 2 and pH 7 for 3 h, the CAC of the samples was measured, as shown in Figure 4d. At pH 2, YF-SDF has the highest CAC (14.66 ± 0.06 mg/g) (*p* < 0.05); at pH 7, BF-SDF has the highest CAC (35.64 ± 0.75 mg/g) (*p* < 0.05), followed by LF-SDF and YF-SDF, while there is no significance different between them. The CAC of SDF at pH 7 in simulated intestinal conditions was greater than that in simulated gastric conditions. This suggests that the conditions in the intestines are more favorable for the attachment of dietary fiber to cholesterol. A high concentration of hydrogen ions in the system during acidic conditions could be the reason. Another point is that under the same pH conditions, LF-SDF, BF-SDF, and YF-SDF have significantly higher CAC than A-SDF and U-SDF, indicating that fermentation can enhance SDF’s ability to adsorb cholesterol. It may be related to the sparse and porous structure and exposed functional groups [42]. The SDFs acquired using the fermentation-assisted extraction technique exhibit superior CAC compared to alternative extraction methods. One research study proved that *Penicillium* YZ-1, which has efficient cellulose-degrading ability, could significantly enhance the CAC of SDFs extracted from grapefruit [43]. Possibly, mixed strain fermentation might be a better choice to acquire SDFs with better CAC abilities, and a recent study has proved that mixed formulations of *Trichoderma viride* and *Aspergillus niger* increased the carbohydrate-associated carbon (CAC) of SDF extracted from navel orange peel by 3.4 times compared to the unfermented group. This was related to the activities of the complex cellulase, i.e., mixed fermentation has stronger activities of carboxymethyl cellulase and β-glucosidase [44].

## 3. Materials and Methods

### 3.1. Materials

White radish was purchased from the local market. All reagents used in this experiment were of analytical grade. For the strain used in the pomace fermentation, *L. plantarum* C11 was isolated from pickled vegetables, *B. amyloliquefaciens* HC2 was isolated from sea cucumber intestines, which were preserved in our laboratory. *W. anomalus* YFJ252 was isolated from pickled radishes and preserved in the China General Microbiological Culture Collection Center (CGMCC No.28267).

### 3.2. SDF Extraction from Radish Pomace

**Figure 5:**
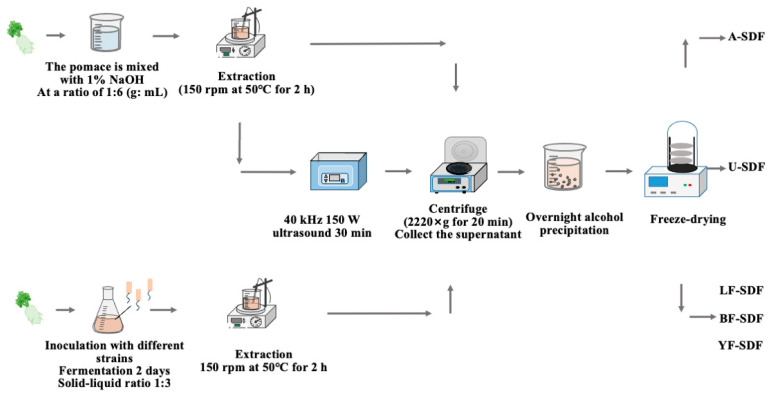
The schematic diagram of the workflow of different extraction methods.

The alkali extraction method, the ultrasound-assisted extraction method, and the fermentation-assisted method were used to extract the SDFs from the radish pomace. The schematic extraction workflow is shown in Figure 5.

#### 3.2.1. Alkali Extraction Method

100 g of white radish pomace was combined with 600 mL NaOH solution (1%, *w*/*v*) at a ratio of 1:6 (g: mL) and incubated at 50 °C, 120 rpm for 2 h; then the mixture was centrifuged (2220× *g*, 20 min). After the centrifugation, the supernatant was collected, and four times greater than the volume of the supernatant of 95% ethanol (*v*/*v*) was added for ethanol participation at 4 °C overnight; the obtained participant was lyophilized and designated as A-SDF.

#### 3.2.2. Ultrasound-Assisted Extraction Method

A total of 100 g of white radish pomace was combined with 600 mL NaOH solution (1%, *w*/*v*) at a ratio of 1:6 (g: mL) and incubated at 50 °C, 120 rpm for 2 h; then the mixture was treated by the ultrasound (40 kHz, 150 W) for 30 min, then performed the same procedure that used to extract A-SDF above, the obtained SDF was designated as U-SDF.

#### 3.2.3. Fermentation-Assisted Extraction Method

The *L. plantarum* C11, *B. amyloliquefaciens* HC2, and *W. anomalus* YFJ252 were activated respectively to prepare a microbial suspension with a 0.8–1.0 OD_600nm_. A certain amount of white radish pomace was weighed and mixed with the water at a ratio of 1:3 (m: V) to make a fermentation culture. The culture also contained 0.2% K_2_HPO_4_, 0.03% MgSO_4_ 7H_2_O, 0.03% CaCl_2_, 0.05% peptone, then 10% (*v*:*v*) of the microbial suspension of different strains was inoculated into the sterilized fermentation culture, and fermented at different conditions. For *L. plantarum* C11 and *B. amyloliquefaciens* HC2 at 37 °C for 48 h, and for *W. anomalus* YFJ252, at 28 °C for 48 h. After fermentation, the SDF were extracted using the same method used for the extraction of A-SDF, and the extracted SDFs were designated as LF-SDF (fermented by *L. plantarum* C11), BF-SDF (fermented by *B. amyloliquefaciens* HC2), and YF-SDF (fermented by *W. anomalus* YFJ252), respectively.

### 3.3. Spectral Analysis

The extracted SDFs were redissolved in ultrapure water (0.1 mg/mL), and their UV spectra were recorded at wavelengths ranging from 200 to 600 nm to detect the purity of the polysaccharides using a UV-visible spectrophotometer (UV-2550, Shimadzu Corporation, Kyoto, Japan) [45]. The lyophilized SDF (1–2 mg) was mixed with KBr (1:100, g:g) and detected by a Fourier transform infrared (FT-IR) spectrometer (Nicolet6700, Thermo Fisher, Waltham, MA, USA) ranging from 400 to 4000 cm^−1^ [46].

### 3.4. Monosaccharide Composition Analysis

The monosaccharide composition of the SDF was analyzed by high-performance liquid chromatography (HPLC) (Agilent1260, Agilent Technologies Inc., Santa Clara, CA, USA) [47]. A total of 5 mg SDF sample was hydrolyzed with 2 mol/L trifluoroacetic acid (TFA) at 100 °C for 6 h. After hydrolysis, the excess acid was removed with methanol, and the samples were vacuum-dried. The vacuum-dried hydrolysis product (100 mg) was then dissolved in 100 μL of 0.3 mol/L NaOH and added to a solution of 120 μL of 0.5 M 1-phenyl-3-methyl-5-pyrazolone (PMP) in methanol, and the reaction was carried out at 70 °C for 1 h, 100 μL of 0.3 mol/L HCl was then added to the mixture, vigorously shaken, and centrifuged at 2400× *g* for 5 min. The mixture was filtered through a 0.22 μm membrane, and 10 μL of the filtrate was injected into a C18 column (Xtimate C18 4.6 × 200 mm 5 μm, Shimadzu LC-20AD, Shimadzu Co., Ltd., Kyoto, Japan) for HPLC analysis. The mobile phase consisted of a mixture of 0.1 mol/L KH_2_PO_4_ (pH 10) and acetonitrile (83:17). The flow rate was set at 1.0 mL/min, and the column temperature was maintained at 30 °C.

### 3.5. Particle Size and Scanning Electron Microscopy (SEM) Analysis

The particle size (nano scale) of the SDF was measured by Brookhaven NanoBrook 90Plus Zeta instrument (Brookhaven Instruments Corporation, New York, NY, USA). The surface of the SEM was observed by field emission scanning electron microscopy (JSM-6390/LV, JEOL Ltd., Tokyo, Japan) at 100× and 1000× magnifications.

### 3.6. X-ray Diffraction(XRD) Analysis

The XRD of the SDFs was obtained using a diffractometer (D8; Bruker, Saarbrucken, Germany) within the scanning range of 10–60° (30 kV, 30 mA).

### 3.7. Differential Scanning Calorimeter (DSC) Analysis

The thermal performance of the SDF was determined using a Differential Scanning Calorimeter (DSC 8000, PerkinElmer, Shelton, CT, USA) [48]. Between 5 to 10 mg of sample was accurately weighed into an aluminum crucible, sealed with an aluminum lid, and an empty aluminum pot with a lid was used as a blank control group. The sample was measured for heat flux intensity change at 10 °C/min within the 20–400 °C temperature range.

### 3.8. Rheological Property Analysis

The viscosity of SDF samples was measured by a rheometer (Thermo Scientific HAAK, Waltham, MA, USA) equipped with a core and plane geometry system (50 mm diameter, 1 mm gap). In the steady shear test, SDF samples’ steady flow behavior with different concentrations (2%, 6%, *w*/*v*) was measured at a 0.1–1000 s^−1^ shear rate range at 25 °C.

### 3.9. Functional Properties

#### 3.9.1. Water Solubility (WS)

Briefly, 0.3 g of freeze-dried SDF was added to distilled water (10 mL) and incubated at 4, 37, and 70 °C for 30 min, respectively. Then the mixture was centrifuged (8880× *g*, 4 °C, 10 min), then the supernatant was dried to a constant weight. The WS was calculated as follows:

(1) $$\mathrm{WS}\;(\%)\;=M_{0}/M_{1}\times 100$$

where *M*_0_ is the weight of the dried supernatant, *M*_1_ is the weight of SDF.

#### 3.9.2. Water Holding Capacity (WHC)

For each test, 0.1 g of the SDF was added to distilled water (3 mL), agitated (37 °C, 2 h), then centrifuged (3200× *g*, 10 min), and the mass of the precipitate was measured. WHC was computed utilizing the formula [49]:

(2) $$\mathrm{WHC}\;(g/g)=\left(M_{2}-M_{0}\right)/\left(M_{1}-M_{0}\right)$$

*M*_0_ is the mass of the centrifuge tube; *M*_1_ is the mass of the centrifuge tube and SDF before centrifugation, and *M*_2_ is the mass of the centrifuge tube and SDF after centrifugation.

#### 3.9.3. Oil Holding Capacity (OHC)

Freeze-dried SDF (0.1 g) was added to 3 mL of soybean oil and agitated at 37 °C for 2 h, then centrifuged at 3200× *g* for 10 min. The mass of the precipitate was measured. OHC was calculated using the following formula:

(3) $$\mathrm{OHC}\;(g/g)=\left(M_{2}-M_{0}\right)/\left(M_{1}-M_{0}\right)$$

*M*_0_ is the mass of the centrifuge tube, *M*_1_ is the mass of the centrifuge tube and sample before centrifugation in grams, and *M*_2_ is the mass of the centrifuge tube and sample after centrifugation.

#### 3.9.4. Glucose Absorption Capacity (GAC)

SDF (5 g) was mixed with 1 L of glucose solution (100 mmol/L) and incubated at 37 °C for 2 h. After that, the mixture was centrifuged at 2220× *g* for 20 min at room temperature. The glucose concentration in the supernatant was measured using the DNS method. The glucose adsorption capacity (mg/g) was calculated using the following equation:

(4) $$\mathrm{GAC}\;(\mathrm{mg}/g)=(C_{1}-C_{2})\;\times \;V/m$$

where *C*_1_ is the initial concentration of glucose (mg/mL), *C*_2_ is the glucose concentration after centrifugation (mg/mL), *V* is the volume of the mixtures (mL), and *m* is the mass of SDF (g).

#### 3.9.5. Inhibition Capacities of α-Amylase

The α-amylase (500 μL, 1 U/mL) and SDF (500 μL, 5 mg/mL) were mixed and incubated at 37 °C for 10 min. Then, 500 μL potato starch solution (1%, *w*/*v*) was added, and the mixture was incubated at 37 °C for 10 min. Then, 1 mL of DNS was added to the mixture and heated in the water bath at 100 °C for 5 min to inactivate the enzyme. Finally, 10 mL of distilled water was added to the mixture. The same concentration of acarbose was used as a positive control. The absorbance (A) of the mixtures was tested at 540 nm. The inhibition rate was calculated through the following equation [50]:

(5) $$\alpha -\mathrm{amylase}\;\mathrm{inhibition}\;\mathrm{rate}\;(\%)=\left[1\right.\left.-\left(A_{3}-A_{4}\right)/\left(A_{1}-A_{2}\right)\right]\times 100$$

*A*_3_ was the absorbance of the mixture, with *A*_1_ without SDF or acarbose, *A*_2_ without α-amylase and SDF or acarbose, and *A*_4_ without α-amylase.

#### 3.9.6. Cholesterol Absorption Capacity (CAC)

An emulsion was prepared by combining 10 g of raw egg yolk with 90 mL of distilled water. Subsequently, 0.5 g of the SDF was combined with 25 mL of emulsion while ensuring the pH was adjusted to 7 and 2 to replicate the conditions in the stomach and intestines. The combination was incubated at a temperature of 37 °C for 3 h. Subsequently, the mixture was centrifuged at 3200× *g* for 10 min to collect the supernatant. The cholesterol content was assessed utilizing a cholesterol determination kit (Sangon Biotech, Shanghai, China). The cholesterol adsorption capacity (mg/g) was calculated using the following formula:

(6) $$\mathrm{CAC}\;(\mathrm{mg}/g)=(C_{0}-C_{1})\;\times \;V/m$$

where *C*_0_ is the cholesterol concentration of the blank group (mg/mL), *C*_1_ is the cholesterol concentration in the experimental group (mg/mL), *V* is the volume of the supernatant (mL), and *m* is the mass of SDF (g).

### 3.10. Statistical Analysis

Each experiment was performed in triplicate independently, and data were designated as mean ± standard deviation (SD). SPSS 20.0 software (SPSS Inc., Chicago, IL, USA) was used for statistical analyses, *p* < 0.05 was recognized as significant.

## 4. Conclusions

In this study, we concluded that white radish is a good source for extracting SDFs. After comparing the alkaline, ultrasonic-assisted, and fermentation-assisted methods, it was found that fermentation-assisted extraction seems the best way to extract white radish SDF, and that the extracted SDFs had better WHC and OHC, and improved GAC and CAC. The study indicated that the modification of the structure and functions of the SDFs extracted through fermentation-assisted extraction is related to the strains used in the fermentation process. BF-SDF has better WS, α-amylase inhibition ability, and CAC (pH = 7). YF-SDF has better WHC, OHC, GAC and CAC (pH = 2), and *W. anomalus* YFJ252 fermented extraction improved the WHC, OHC, GAC and CAC (pH 2 and pH 7) by 3.06, 1.65, 3.19, 1.27, and 1.83-fold, respectively than the SDF extracted through alkaline extraction. It has been proved that white radish SDF could be used as a functional food ingredient.

## Figures and Tables

**Table 1:** The monosaccharide composition of the SDFs.

Monosaccharides (%)	A-SDF	U-SDF	LF-SDF	BF-SDF	YF-SDF
Mannose	0.47	0.66	0.34	0.55	4.49
Ribose	0.19	0.24	0.25	0.32	0.38
Rhamnose	2.28	3.39	4.28	5.31	4.73
Glucuronic acid	0.15	0.38	0.19	0.27	0.24
Galacturonic acid	10.21	17.84	8.37	12.99	10.30
Glucose	2.13	3.64	2.01	3.09	2.57
Galactose	2.25	3.84	2.41	2.89	2.33
Xylose	0.13	0.22	0.07	0.11	0.15
Arabinose	3.30	5.61	2.77	3.58	3.09
Fucose	0.37	0.53	0.44	0.48	0.42

## Data Availability

Data are contained in the article.

## Supplementary Materials

The following supporting information can be downloaded at: https://www.mdpi.com/article/10.3390/molecules29020500/s1, Figure S1: HPLC analysis of the SDFs.
