# Supplementary figures and images for: Characterization and Function Analysis of Soluble Dietary Fiber Obtained from Radish Pomace by Different Extraction Methods
Source: Molecules. 2024 Jan 19;29(2):500. doi: 10.3390/molecules29020500 (PMC10818875; doi:10.3390/molecules29020500)

Supplementary Materials:

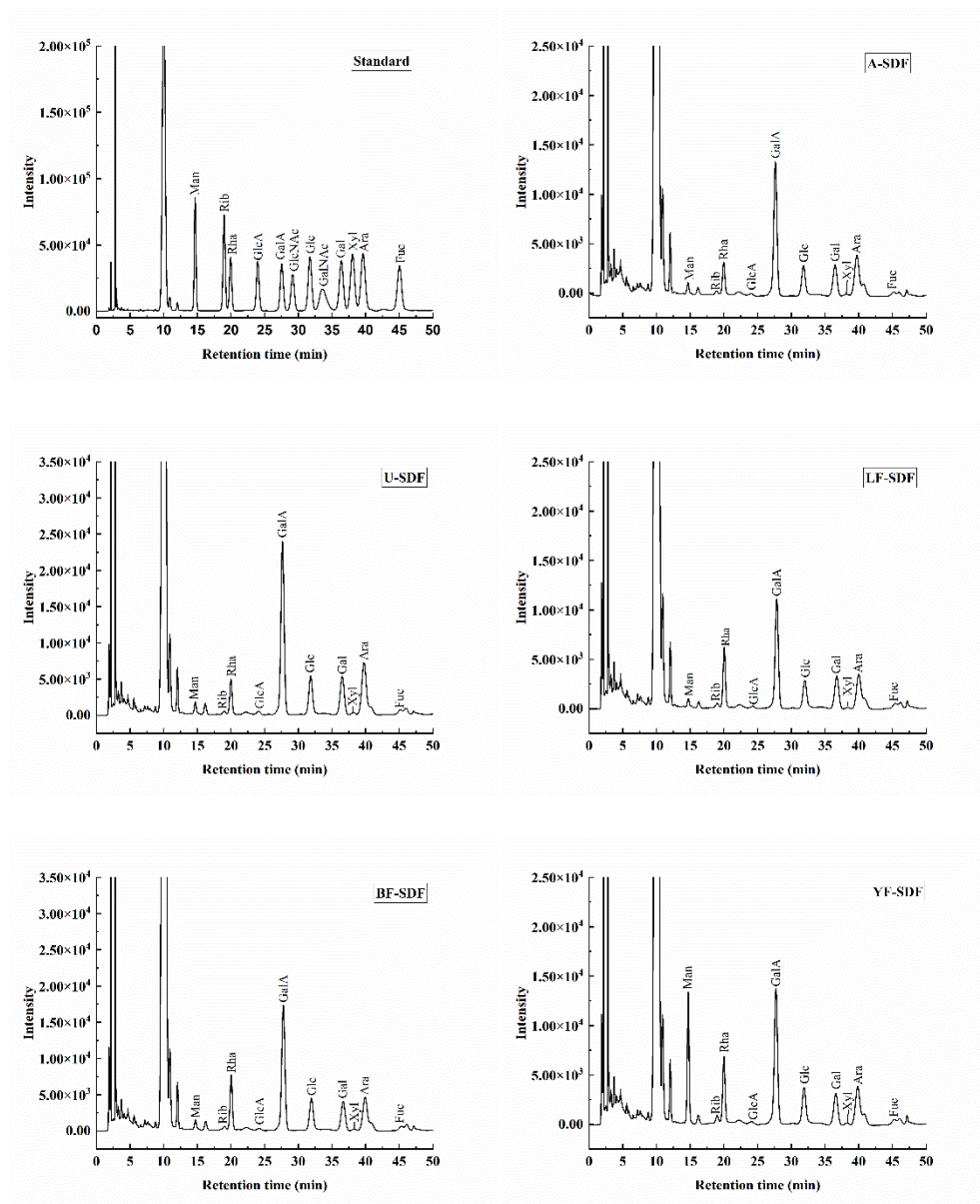

Figure S1: HPLC analysis of the SDFs.

Supplement: Supplementary file 1 [file molecules-29-00500-s001.zip › molecules-2801545-supplementary.pdf]
